# Plant-derived cis-β-ocimene as a precursor for biocompatible, transparent, thermally-stable dielectric and encapsulating layers for organic electronics

**DOI:** 10.1038/srep38571

**Published:** 2016-12-09

**Authors:** Kateryna Bazaka, Ryan Destefani, Mohan V. Jacob

**Affiliations:** 1 School of Chemistry, Physics, and Mechanical Engineering, Queensland University of Technology, Brisbane, QLD 4000 Australia; 2 Electronics Materials Lab, College of Science and Engineering, James Cook University, Townsville, QLD 4811 Australia; 3 Institute of Health and Biomedical Innovation, Queensland University of Technology, Brisbane, QLD 4000, Australia; 4 Institute for Future Environments, Queensland University of Technology, Brisbane, QLD 4000, Australia; 5 CSIRO−QUT Joint Sustainable Materials and Devices Laboratory, Commonwealth Scientific and Industrial Research Organisation, P.O.Box 218, Lindfield, NSW 2070, Australia

## Abstract

This article presents low-temperature, one-step dry synthesis of optically transparent thermally-stable, biocompatible cis−β−ocimene-based thin films for applications as interlayer dielectric and encapsulating layer for flexible electronic devices, e.g. OLEDs. Morphological analysis of thin films shows uniform, very smooth (*R*_*q*_ < 1 nm) and defect-free moderately hydrophilic surfaces. The films are optically transparent, with a refractive index of ~1.58 at 600 nm, an optical band gap of ~2.85 eV, and dielectric constant of 3.5−3.6 at 1 kHz. Upon heating, thin films are chemically and optically stable up to at least 200 °C, where thermal stability increases for films manufactured at higher RF power as well as for films deposited away from the plasma glow. Heating of the sample increases the dielectric constant, from 3.7 (25 °C) to 4.7 (120 °C) at 1 kHz for polymer fabricated at 25 W. Polymers are biocompatible with non-adherent THP–1 cells and adherent mouse macrophage cells, including LPS-stimulated macrophages, and maintain their material properties after 48 h of immersion into simulated body fluid. The versatile nature of the films fabricated in this study may be exploited in next-generation consumer electronics and energy technologies.

Among polymer-based devices, OLEDs are attracting significant attention as an alternative for current display technology in applications such as televisions and mobile phones, as well as a highly efficient lighting solution^1^,^2^,^3^. OLEDs have also emerged as a promising technology for the effective and rapid delivery of health care, through applications such as affordable point-of-care immunobiosensors with sensitivity that approaches lower limit of detection of typical clinical laboratory instrumentation^4^, or as miniature implantable devices for continuous monitoring in patients with chronic conditions. Benefits from the unique advantages offered by organic materials, biointegration is a rapidly growing field of research that aims to revolutionise the way biomedical services and medical care are delivered to patients. Advanced applications of OLEDs include biophotonics and optogenetics^5^, where OLEDs are used for highly-resolved spatiotemporal optical stimulation of cells and organisms to modulate their activity *in vitro* and *in vivo*^6^.

To enable the necessary level of integration with neurological or physiological circuitry, or desired sensitivity for optical sensing and light therapy, a combination of biocompatibility, performance and stability under aqueous conditions are required. Fabrication of OLED devices requires multiple layers responsible for the transfer and recombination of electrons which emit light through electroluminescence. Current OLEDs have a relatively low level of efficiency with an uneven ratio of electrons to holes passing through anodes and cathodes which have mismatched charge transport properties preventing effective recombination^7^. Key to its design, a dielectric layer, e.g. SiO_2_ or poly(methylmethacrylate), can be used to significantly improve efficiency by controlling the flow of holes and electrons for improved charge density. Further gains in OLED efficiency can be made through the use of such charge transport controlling layers as hole-transporting polyaniline–camphorsulfonic acid^8^ or polypyrrole–dodecylbenzene sulfonic acid^9^ that balance injection and accumulation of charge carriers in the device.

However, the majority of currently employed organic precursors used to fabricate charge transport controlling layers are synthetic, non-renewable and may degrade to potentially harmful products when exposed to aqueous conditions^10^,^11^. Whereas most methods for fabrication of environmentally-friendly, biocompatible electronic-grade have focused on solution processing^12^,^13^,^14^,^15^, solvent-free plasma-enhanced deposition of essential oil-based polymer thin films was demonstrated by our group^10^,^16^,^17^. With the charge transport properties similar to those of conventional materials, essential oil-based films are smooth and defect-free. Depending on the chemistry of the precursor and processing conditions, the transport properties of these films vary from a complete insulator, e.g. linalyl acetate thin films^18^, to electron-blocking hole-transporting polyterpenol films that can control the flow of charge in OLEDs^10^,^19^.

This paper investigates the dielectric behaviour of another natural precursor, cis−β−ocimene, in the 1 Hz to 1.2 PHz frequency range with the intent to apply these films as a dielectric interlayer and encapsulating layer in organic electronic devices. Biocompatibility of these films with mammalian cell lines is also investigated to assess their suitability for biomedically-relevant devices.

## Methods

The dielectric properties of thin films derived from cis−β−ocimene were investigated as a function of frequency, polymer fabrication parameters (deposition power level and substrate location), and temperature. As substrate materials, glass slides, quartz slides and KBr windows were employed to be used for ellipsometric, FTIR and wettability studies. Metal–insulator–metal (MIM) structures were fabricated for impedance spectroscopy measurements. Prior to plasma deposition, the glass slides were washed in a solution of Extran and distilled water, ultrasonically cleaned for 30 min and rinsed with isopropanol to remove any inorganic contaminants. The glass slides were then thoroughly rinsed with double distilled water and blown dry using N_2_.

### Polymer synthesis

**Figure 1:**
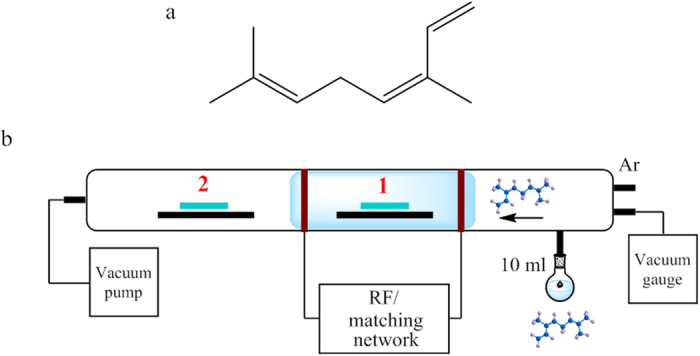
(**a**) Chemical structure of cis−β−ocimene. (**b**) Custom-made polymerisaiton reactor. Polymerisation took place within (position 1) and downstream from the plasma glow region (position 2).

For all samples, 99.8% pure cis−β−ocimene (Australian Botanical Products Ltd.) was used without further modification (Fig. 1a). For each deposition, 10 mL of cis−β−ocimene was placed into monomer flask. Thin films were deposited using plasma deposition in a cylindrical glass reaction chamber (Fig. 1b). Briefly, an RF generator delivered radio frequency (RF) power at 13.56 MHz to the chamber via capacitively coupled copper electrodes, spaced 0.1 m apart and 0.05 m from the monomer inlet. The chamber was evacuated to 10 Pa, at which point the plasma discharge was initiated and allowed to stabilise for 1−2 min. Then, the monomer was released into the chamber. Deposition took place within the pressure range of 20−40 Pa (depending on the input power level used). Films were fabricated at the input power levels of 25 W, 50 W and 75 W, with substrate located either within the plasma glow (indicated as position 1 on Fig. 1b) or downstream from the plasma glow (position 2 on Fig. 1b). The latter was done to investigate the effect of in-plasma ion bombardment on the physico-chemical and electrical properties of the resultant thin film material. Monomer flow rate was controlled using a vacuum stopcock on the inlet. Prior to deposition, the chamber was flushed with Ar to provide an oxygen-free atmosphere. Time of deposition was varied to obtain films of the desired thickness (200−2000 nm).

### Polymer characterisation

Optical properties of the films were derived from data acquired through ellipsometry (M-2000, J.A. Woollam Co., USA) at three different angles of incidence (φ = 55°, 60°, and 65°) over the wavelength range of 200–1000 nm by means of regression analysis (WVASE32 software package). The angles of incidence were selected to be near experimentally-determined Brewster angle of the polymer film to ensure the maximum difference between an *s* and a *p* component of the polarization state of the light incident upon the polymer film. Thickness was estimated by first applying Cauchy dispersion to the data under the assumption that the films are optically transparent above 400 nm (as evident from transmission data) and homogenous. A surface roughness layer above the Cauchy layer was used to refine the model. Optical properties (real refractive index, *n*, and extinction coefficient, *k*) across the sampled wavelength were then estimated by fitting Gaussian or Lorentz harmonic oscillators to the data. A mean squared error (MSE) value below 2 was observed for all measurements.

Atomic force microscopy (AFM) was employed to determine the surface roughness characteristics of the films. These measurements were performed using an Ntegra Prima (NT-MDT, Russian Federation) atomic force microscope scanning with constant force at a maximum resolution of 100 × 100 μm. Semi-contact (tapping) mode was used to limit sample damage from lateral movement of the cantilever tip. Surface wettability studies were performed using a contact angle system (KSV 101, CCD camera) employing the sessile drop method following the method reported previously^20^,^21^.

### Thermal Study

Thermal stability of optical properties of cis−β−ocimene polymer thin films was performed *in situ* using the ellipsometry heating stage. Measurements were collected in the 25−405 °C temperature range, with the interval of 10 °C. For a molecular composition analysis samples were heated to 100 °C, 200 °C and 300 °C with a temperature-controlled hotplate, leaving the samples at the set temperature for at least 5 min to ensure uniform heating. Fourier transform infrared spectroscopy (FTIR) was then performed on these samples using 100FT-IR spectroscope (Perkin Elmer, USA) in attenuated total reflection (ATR) mode. The ATR data was then translated into transmittance data using instrument software correction function.

### Device fabrication

Aluminium electrodes were first deposited onto clean glass slides (25 mm × 75 mm) using thermal evaporation (a Hivac thermal evaporation unit). Al wire (99.9% pure) was placed within the Al_2_O_3_-coated tungsten boat. The system was then evacuated to a pressure of 20 Pa, at which stage the plasma was initiated for 1 min to clean the surface of the substrate. Subsequently, the pressure was allowed to reach <0.005 Pa, at which point the current was passed through the boat and Al began to melt and evaporate. Metal films of ~100 nm were used in this study. Using a shadow mask, a polymer coating was deposited following the synthesis procedure described above, followed by the thermal evaporation deposition of 5 mm × 10 mm top electrodes. Copper wires were attached to the electrodes using CircuitWorks two–part silver conductive epoxy to enable interfacing with measurement device.

### Device characterisation

The dielectric function of the devices was analysed between frequencies of 100–100 kHz using a Hioki 3522 LCR meter (Hioki, Japan). The impedance *Z*, capacitance *C* and phase angle *θ* were measured with no applied DC bias. From the known device surface area (approximately 50 mm^2^) and measured capacitance values, real *ε*_*r*_ and imaginary *ε*_*r*_’ parts of the dielectric constant of the polymer film were determined as *ε*_*r*_ = *dC*/*A*ε_0_ and *ε*_*r*_’ = *ε*_*r*_ *tanδ*, where *d* is the thickness of the film, *A* is the cross section area and ε_0_ is the permittivity of free space. Keithley 2636 A source meter was used for current−voltage measurements that were taken between 0 V and 200 V, with a step size of ~1 V. The voltage was applied as a staircase function, with holding time of 50 ms per point. Since high initial current is observed when a voltage step is applied to the capacitive MIM structure, multiple measurements per each applied voltage were taken to obtain a realistic value of the leakage current. Current density was calculated by dividing the final point of each transient by the area of MIM. Since the resistivity of the cis−β−ocimene samples was found to be high (~GΩ) while the resistance of the experimental apparatus was found to be very small (~Ω), separation of the contact and spreading resistances were therefore unnecessary and accurate measurements were obtained using the two-probe measurement technique.

The dependence of the dielectric constant on temperature was found using an LCR meter measuring the capacitance along the frequency range of 1 Hz−100 kHz during heating in 10 °C intervals from 25 °C to 120 °C.

### Polymer biodegradation and biocompatibility

Stability in aqueous environment was evaluated by immersing polymer thin films coated onto glass substrates into simulated body fluid comprising of Na^+^ 142.0 mM, K^+^ 5.0 mM, Mg^2+^ 1.5 mM, Ca^2+^ 2.5 mM, HCO^3−^ 4.2 mM, Cl^−^ 147.8 mM, HPO_4_^2−^ 1.0 mM and SO_4_^2−^ 0.5 mM, and incubating them for 2 days at 37 ± 1 °C 22. After incubation, the samples were gently rinsed with double-distilled water and allowed to air dry. Their optical, surface and chemical properties were then evaluated using ellipsometry, AFM and FTIR, respectively.

For biocompatibility studies, human acute monocytic leukemia (THP–1) cells were cultured in the presence of polymer-coated glass slides (0.5 mm thick cover slip), with unmodified glass slides serving as control. Polymer coatings were synthesised following the standard protocol at 25 W, 50 W and 75 W. Sterile polymer-coated and uncoated samples were then placed into 24-well plates. THP–1 cells were suspended in RPMI–1640 growth medium containing 100 IU/ml penicillin, 100 IU/ml streptomycin, with 2% (v/v) 15 μg/ml L-glutamine, 10% (v/v) HI–FBS, and 2% (v/v) HEPES. The THP–1 cells were then incubated at 37 °C with 5% CO_2_ in humidified atmosphere until desired cell density was attained. At this point, the THP–1 cells were harvested from the growth flasks, centrifuged and resuspended in the fresh media for plating. Cells were seeded at density of 5 × 10^5^ cells per ml in 24-well plate. The experiment was repeated three times, with the minimum of three samples for each sample type tested per experiment iteration. RPMI medium was used as negative control, and RPMI seeded with the THP–1 cells was used as a positive control. Cytotoxitiy and proliferation of the THP–1 cells was assessed at 24 and 48 hours of incubation using dye exclusion method. Trypan blue solution was used to differentiate between healthy viable and damaged or dead cells. Optical microscopy (BH2 Olympus, Center Valley, PA) fitted with a QImaging camera was used to visualise and count the cells. Biocompatibility was assessed with respect to cell viability and proliferation.

To evaluate contact compatibility of polymer coatings and cell-surface interactions, mouse macrophage cells were allowed to attach to the polymer-modified and unmodified glass slides, and their adhesion, shape and viability assessed after 24 h and 48 h of incubation. Cells were kindly provided by Infectious Diseases and Immunopathogenesis Research Group, James Cook University, where they were harvested with approval from James Cook University Animal Ethics Committee and in accordance with the Australian Code of Practice for the Care and Use of Animals for Scientific Purposes (National Health and Medical Research Council)^23^. Prior to inoculation, cells were resuspended in RPIM medium to achieve the density of 5 × 10^5 ^cells per ml, and added to 24–well plate containing unmodified and polymer-coated glass samples. After 24 h of incubation, culture media was replenished, and cells were allowed to incubate for further 24 h. For visualisation, macrophage cells that failed to attach were removed by first aspirating the media, followed by a gentle wash with PBS three times. Diff-Quik staining system was used to stain the cells. Their morphology and attachment preferences examined using a light microscope.

Data were analysed using SPSS 16.0 statistical package. All values were reported as the mean ± SD. Differences between groups were analysed by the separate variance estimation *t*–test and analysis of variance (ANOVA). The value of *p* < 0.05 was defined as statistically significant.

## Results and Discussion

The potential of organic polymer thin films to enable next-generation high-performance affordable organic electronics is well established^24^. Among frequently sighted benefits are their versatility, where they can be applied as a conducting, insulating or semiconducting layer, mechanical flexibility and weight reduction^16^,^25^. Their lower production cost when compared to inorganic counterparts, e.g. silicon, makes them highly attractive for commercial photovoltaics and electronics, with many organic field effect transistors (OFET), organic solar cells (OSC) and organic light emitting diodes (OLED) penetrating the market^26^,^27^,^28^. Furthermore, polymer thin films can be fabricated from naturally occurring precursors using synthesis methods that are more energy and material efficient and environment- and human-health-friendly^11^. There is also a significant potential to design polymer thin films that are inherently biodegradable, hence eliminating the need for post-use waste management and potentially enabling such devices as non-retrievable environmental or implantable sensors.

With the growing awareness of environmental cost associated with not only device operation but also production of electronic materials, several very promising environmentally-friendly, biocompatible electronic-grade solution-processed materials have been developed, including natural resin shellac^12^, silk fibroin^13^, deoxyribonucleic acid, chicken albumen^14^, glucose^15^, and others. Solvent-free, dry plasma-enhanced chemical vapour deposition of essential oil-based polymer thin films was demonstrated by our group^10^,^16^,^17^. Low-pressure, low-temperature plasmas are a dynamic environment that comprises highly reactive chemical species, energetic photons, and electric fields. The interplay between these effects is dependent on the physical state and chemical composition of the precursor and the processing parameters, such as applied power and precursor residence time, and in turn determines the nature of the material produced in this plasma environment.

### Direct deposition

In the high-throughput fabrication of thin film-based electronics, the deposition rate at which quality thin film can be deposited is of notable significance. In general, plasma polymerisation is a competitive process where polymer formation is competing against etching and desorption. The deposition rate is known to depend upon the energy invested per particle of gas mixture flowing through the glow discharge zone. Hence, for a given deposition assembly this is a function of several process parameters, including pressure, applied power, flow rate, and monomer species^29^. Beyond these parameters, it is known that substrate thermal and energetic conditions also influence the plasma polymerisation processes, including adsorption, desorption, diffusion, and chemical reactions. These substrate conditions are themselves a function of the energy per bombarding particle, and the flux density of these particles^30^. Higher energy dose per monomer molecule leads to increased substrate heating by way of radiative processes and chemical reactions at the surface, which in turn negatively correlates with deposition rate beyond a certain temperature threshold^31^. This could indicate that an adsorption-desorption equilibrium is beginning to become a rate limiting process within the deposition at higher powers (and thus substrate temperatures).

Deposition rates for plasma polymers derived from non-synthetic sources have been shown to demonstrate both a positive correlation with power (e.g., γ-terpinene^32^) and a negative correlation with power (e.g., linalool^26^). Decreasing deposition rate at higher applied power could be attributed to a shift in the balance between deposition and etching activities, with higher powers favouring the latter^33^. Furthermore, it is known that below certain energy per molecule threshold, the rate of plasma polymerisation is strongly dependent upon the structure of the feed gas (or monomer). At higher energy per monomer molecule, the structure of the feed gas exerts less influence on the rate of deposition owing to increased monomer fragmentation^34^. In the case of cis−β−ocimene, the deposition rate was found to increase with applied power, from 95 nm/min at 25 W to 206 nm/min at 75 W. Below 10 W, the deposition rate was significantly lower, indicated limited monomer fragmentation and polymer formation. This agrees well with the established relationship between the plasma energy density and the dissociation of reactant gases^35^. Thickness mapping across the substrate showed uniform deposition.

### Polymer characterisation

**Figure 2:**
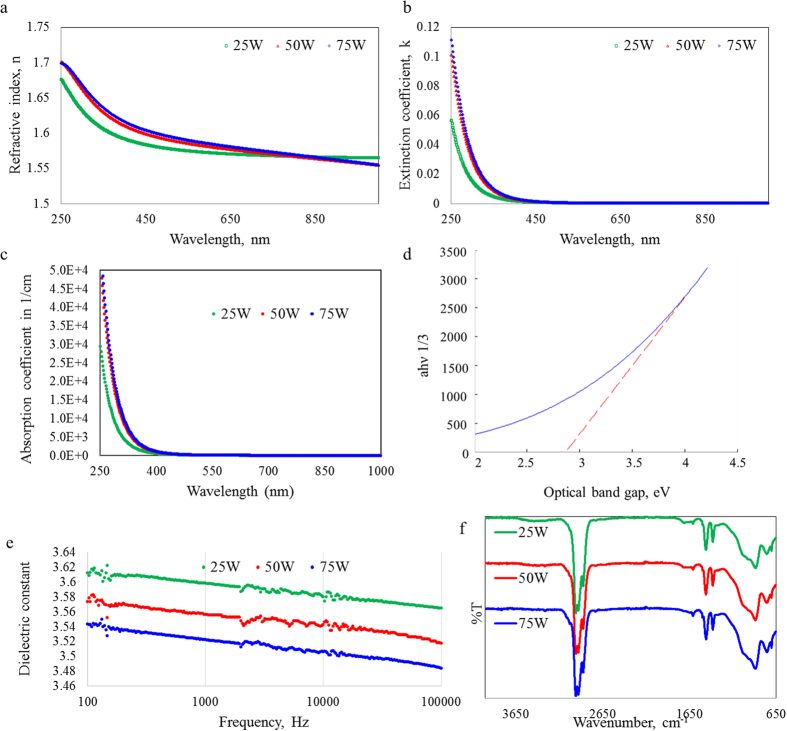
Effect of applied RF power on the optical and chemical properties of cis−β−ocimene polymer thin films:(**a**) refractive index, (**b**) extinction coefficient, and (**c**) absorption coefficient. (**d**) Linear fit to absorption data for polymer thin film fabricated at applied power of 25 W showing band gap. (**e**) Dielectric constant as a function of deposition power measured using an LCR meter across 100−100 kHz frequency range. (**f**) FTIR spectra for cis−β−ocimene thin film deposited at different applied power.

The modelled optical properties of the films are shown in Fig. 2. The films are optically transparent, with the extinction coefficient approaching zero in the visible wavelength range (400−700 nm). Below 700 nm, the refractive index was found to increase as a function of deposition power, with the difference most evident for wavelengths in the 250−450 nm region. This can be attributed to an increase in density of the films at higher power levels. As power level increases so does the intensity of ion bombardment and the flux of ions to the growing layer^36^. This results in a higher refractive index due to the increased crosslinking in the films, which is consistent with results previously reported for films fabricated from other secondary plant metabolites, e.g. terpinene-4-ol, linalyl acetate, cineol, and others^37^. For wavelengths above 400 nm, the refractive index is characteristic of a normal dispersive medium, and is approximately 1.57.

Through the use of the Tauc-Lorentz oscillator model the absorption data (Fig. 2d) was fitted to determine the optical band gap of the films, yielding *E*_*g*_ (25 W) of 2.86 eV, *E*_*g*_ (50 W) of 2.83 eV, and *E*_*g*_ (75 W) of 2.82 eV. Optical absorption in the film occurs as photon energy absorption by the electrons where energy is conserved^38^. Photon absorption excites electrons from a full to an empty band causing an increase in the absorption coefficient *α*(*ν*). The onset of this sudden change in absorption coefficient is represented by the fundamental absorption edge and the optical band gap E_g_ represents the corresponding energy. Numerous values of *n* were tested to understand the nature of the transition. The results were plotted against energy hν and the band gap was estimated by extrapolating the linear region of the curve to the photon energy axis, where the intercept gave the value for the energy gap. For amorphous materials, a value for *n* of 2 is typically chosen indicating a parabolic function for the density of states distribution^39^. This value resulted in the best linear fit demonstrating that indirect transition occurs in the films^40^. Plasma polymers have a characteristically high level of disorder allowing the value of the gap to diverge from the energy gap value by the width of the range of localised states in the valence and conduction band^41^.

The differences in optical band gap with RF deposition power may be in part attributed to varying sp^2^ carbon content and hydrogen content in the polymer^42^. The obtained band gap values can be classified as either semiconducting or insulating. Considering the highly amorphous nature of films fabricated with plasma polymerisation and their characteristically low mobility, these films are being regarded as insulators^29^. Therefore they are proposed for insulating applications such as the dielectric layer in OLEDs. At higher power levels the crosslinking induced in the film increases its density thereby reducing the amount of light which can pass through it. This increases the extinction coefficient and the film absorption resulting in the trend found for the optical band gap.

The insulating nature of the polymer thin films was confirmed using LCR measurement. Figure 2(e) shows the dielectric constant across the measured frequency range. The noise seen in the data is attributed to the change in the measurement range, and is not characteristic of the polymer films themselves. All the samples have approximately the same frequency dependence on the dielectric constant. The deposition process induces a large amount of cross linking resulting in a large reduction in electron mobility. Higher power levels increase the degree of cross linking and results in a more compact structure, decreasing the leakage current density and dielectric constant^16^. The higher dielectric constant in the low frequency regions has previously been reported for other thin films deposited through plasma polymerisation^43^. The formation of depletion regions and interfacial polarisations can occur at the insulator to metal junction due to the inefficiency of charge migration under an electric field at lower frequencies^16^. At higher frequencies these effects are much less pronounced and therefore do not have a significant effect on the dielectric constant.

The current−voltage characterisation was used to determine the charge transport mechanism of the material and the conductivity. The current density−voltage (*J−V*) curves for devices containing cis−β−ocimene polymers fabricated at different applied power conditions showed that the current density was dependent on the applied field, showing a sharp increase in *J* above ~70 V and increasing by ~ three orders of magnitude at the applied field of 200 V. The dc conductivity, calculated as *σ*_dc_ = *Jd/V*, where *d* is the thickness of the cis−β−ocimene, and *J* and *V* are the measured current density and applied voltage, respectively, was also dependent on the applied field. At 200 V, the maximum conductivity for all films was recorded, ranging in 10^−12^–10^−11 ^Ω^−1^ m^−1^. The conductivity was lower for polymers fabricated at high applied power. At high frequencies, the motion of charge carriers along polymer chains contributes to the conductivity, whereas defects and inter-chain charge transport reduce electrical conductivity. The conductivity of cis−β−ocimene films was significantly lower compared to other amorphous, highly cross-linked polymers deposited using plasma, e.g. plasma-deposited thiophenes or polyanilines that displayed *σ* of 10^−4^–10^−8 ^Ω^−1^ m^−1^ and 10^−6^–10^−8 ^Ω^−1^ m^−1^, respectively^44^,^45^. From the device perspective, this means that devices containing cis−β−ocimene dielectric films would experience relatively low leakage currents through the material. Independent of film thickness and deposition power, the field at which breakdown occurred, *F*_b_ was above 1.2 MV cm^−1^, which is comparable to that reported for plasma-deposited polypyrrole, at 1.5 MV cm^−1^ 43, linalyl acetate, at 1.8 MV cm^−116^, or octafluorocylcobutane, at 2 ± 0.5 MV cm^−1^ 46. It has previously been shown that the F_b_ of plasma-deposited thin film, in that instance benzene, can be significantly enhanced through fine-tuning of deposition parameters, e.g. where monomer is introduced into the deposition reactor, deposition rate and deposition pressure, etc., which presents an opportunity to further optimise the *F*_b_ of cis−β−ocimene films^46^.

The charge transport was determined by comparing the theoretical value of the field-lowering coefficient *β* with experimental value of *β* following the procedure outlines in detail in^47^. In insulating film, such as that deposited from cis−β−ocimene, typical mechanisms to consider include Richardson−Schottky transport, Poole−Frenkel transport and space charge limited conduction. Briefly, Richardson−Schottky transport involves lowering of the potential barrier that arises from the energy level misalignment of the dielectric and metallic electrode materials by applied field. Poole−Frenkel transport involves lowering of the trap barrier in the bulk of an insulator. Space charge limited conduction describes a mechanism where carrier injection into the cathode results in an excess carrier density at the metal-insulator interface and a space charge region. Theoretical values of *β*_RS_ and *β*_PF_ were calculated to be 3.25 × 10^−5^ and 6.49 × 10^−5^, respectively, whereas the experimental values for cis−β−ocimene films were estimated to be 1.2−1.4 × 10^−5^. The values of *β*_exp_ best agree with the theoretical *β*_RS_, suggesting that in the context of a field activated mechanism, Richardson−Schottky transport dominated. It should be noted that given the observed thickness-dependent conductivity, space charge effects may also be present.

The amorphous, highly cross-linked nature of polymers fabricated from cis−β−ocimene was confirmed by FTIR analysis (Fig. 2f). A spectral interpretation of the data was performed via analysis of the vibrational group frequencies as shown in Supplementary Table S1. The characteristic functional groups present in the films include primarily methyl and methylene groups as well as hydroxyl, alkene and carbonyl functionalities. The hydroxyl group in the films introduces the possibility of a large presence of hydrogen bonds in the sample which could be intramolecular or intermolecular. The band is very broad and covers a relatively large number of frequencies indicating it is more likely to be responding to an intermolecular presence however either configuration is plausible. Independent of applied power, the most dominant peaks measured indicate the majority of the sample consists of methyl and methylene groups. This is expected as the monomer itself largely consists of hydrogen and carbon atoms. The combination of strong methyl and methylene presence together with a weaker methyl band are generally representative of regularity for the linear backbone structure^48^. However this is a film created by plasma polymerisation and will inherently have a high level of disorder. The peak associated with −OH group appears to be more prominent in samples fabricated at lower power levels.

A peak which does not appear very clear in the FTIR data at room temperature but does become more prominent after heating is at wavenumber 1706 cm^−1^. This peak generally appears between wavenumbers 1700−1725 cm^−1^ and is attributed to the presence of a carbonyl group, specifically a carboxylic acid. A very small peak is visible at wavenumber 1603 cm^−1^ indicating C=C vibration, suggesting the presence of conjugated doubled bonded carbons. The original monomer contains conjugated carbon double bonds and therefore it is plausible that some of this is maintained and embedded in the structure of the polymer. Peaks across the lower wavenumbers demonstrate skeletal C−C vibrations. It is expected that fundamentally this polymer consists of carbon chains which are either cross linked by carbons bonds to nearby chains or terminated with hydrogen atoms. Therefore vibrations at these frequencies are anticipated.

Presence of polar groups, such as −OH, in the films deposited at lower applied power affected the solvent−surface dynamics. Droplet formations on a flat planar surface are governed by the balance of the adhesive and cohesive forces inside and between the droplet and the surface. Adhesive forces act to disperse and spread the droplet over the surface while cohesive forces try to draw the liquid together to counteract spreading. This behaviour is described by Young’s equation, which relates the contact angle to the surface free energy of the three phase system containing a solid, liquid and vapour phases. The manner in which surfaces interact with liquids is critical for both solution-processed electronics and biomedical applications.

All water contact angles were estimated to be ~80° indicating the films are moderately hydrophilic. Moderate hydrophilicity is generally desired for biomedical applications, where it is associated with improved biocompatibility, cell attachment, and proliferation^49^. Increasing applied power resulted in surfaces that were slightly more hydrophobic; however, the difference was not statistically significant. Examination of the contact angle evolution with time revealed slight decrease of the measured contact angle within first 30 s, however, once the equilibrium contact angle was reached, the contact angle remained stable. The initial decrease in contact angle can be attributed to the reorientation of polymer chains, which tends to decrease with an increase in the degree of cross-linking, brought upon by an increase in the degree of monomer dissociation under high applied power conditions. After 30 s, the stability of the contact angle (observed for >5 min) suggests chemical stability of the film in contact with water.

By using a combination of different liquids, the surface properties of the material can be characterised in terms of several parameters, including total surface energy and its Van der Waal, electron donor and electron acceptor components (Table 1). Based on these values, the expected behaviour of the films in contact with such solvents as chloroform and dichlorobenzene was examined. These solvents are widely used in the deposition of organic semiconducting layers from solution^50^,^51^,^52^,^53^. Independent of applied power used during film deposition, these solutions are expected to completely wet the surface (with estimated contact angle of 0°). This makes the surface of the film a suitable substrate for spin coating, drop coating, doctor blading and inkjet printing manufacturing applications, where good adhesion between layers is required^54^. Complete wetting by these solvents was confirmed experimentally, although it was not possible to measure the contact angle using the available capture system.

**Figure 3:**
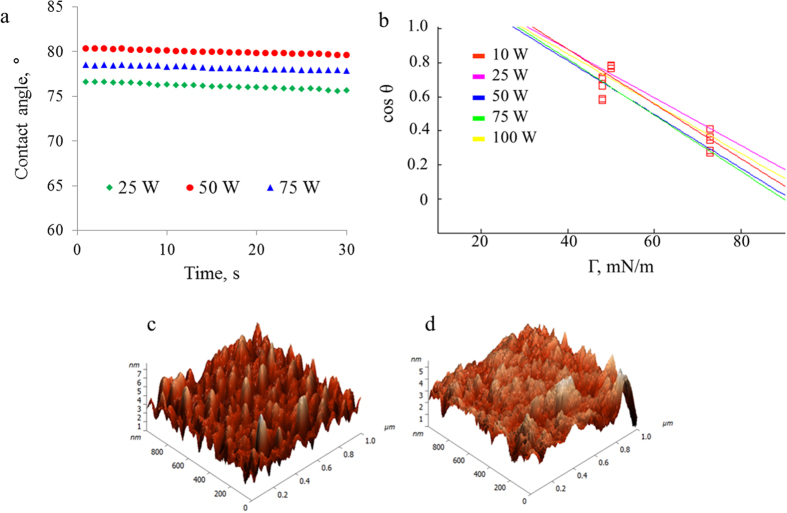
(**a**) Evolution of water contact angle on the surfaces of cis−β−ocimene polymers deposited at different applied power. (**b**) Zisman plot of polymers fabricated at different applied power obtained using water, ethylene glycol and diiodomethane as test liquids. (**c,d**) Representative atomic force microscopy images of cis−β−ocimene polymers fabricated at (c) 25 W and (d) 75 W applied power. Scanning area of 1 × 1 μm.

In addition to surface chemistry, wettability can be related to surface roughness by Wenzel equation^55^. A topographic analysis performed on samples fabricated under different applied power conditions confirmed that irrespective of the applied power, all films were smooth and pinhole free. AFM measurements were taken at two resolutions (1 × 1 μm and 10 × 10 μm), performed due to roughness being a length-scale parameter, and the representative images are shown in Fig. 3. All polymer films were found to be smooth and pinhole free. The roughness parameters were quantified and are summarized in Table 1. A reduction in roughness with increasing power is attributed to a higher ion bombardment at these power levels which has been shown to etch the surface^56^. At 10 × 10 μm, the skewness is a small positive value indicating the films contain more peaks than valleys. The increased kurtosis at higher resolution (1 × 1 μm) occurs as this coefficient is sensitive to isolated peaks and valleys, more so than skewness^57^.

### Remote deposition

In addition to deposition that takes place within the plasma region, certain conditions favour transport of active species and energy to remote sites away from the active region. The major advantage of this deposition method lies in the fact that the plasma creation and application regions are separated. This minimises potentially-deleterious effects of direct ion bombardment, and may be conducive to incorporation and/or retention of the biologically-relevant chemical functionalities into the growing film structure. However, this is generally achieved at the expense of the deposition rate, with rate decreasing sharply with distance from the active plasma region^58^. This was the case for cis−β−ocimene deposition, where deposition rate decreased from 159 nm/min to 94 nm/min as the substrate was moved downstream from the plasma region (at 50 W applied power).

**Figure 4:**
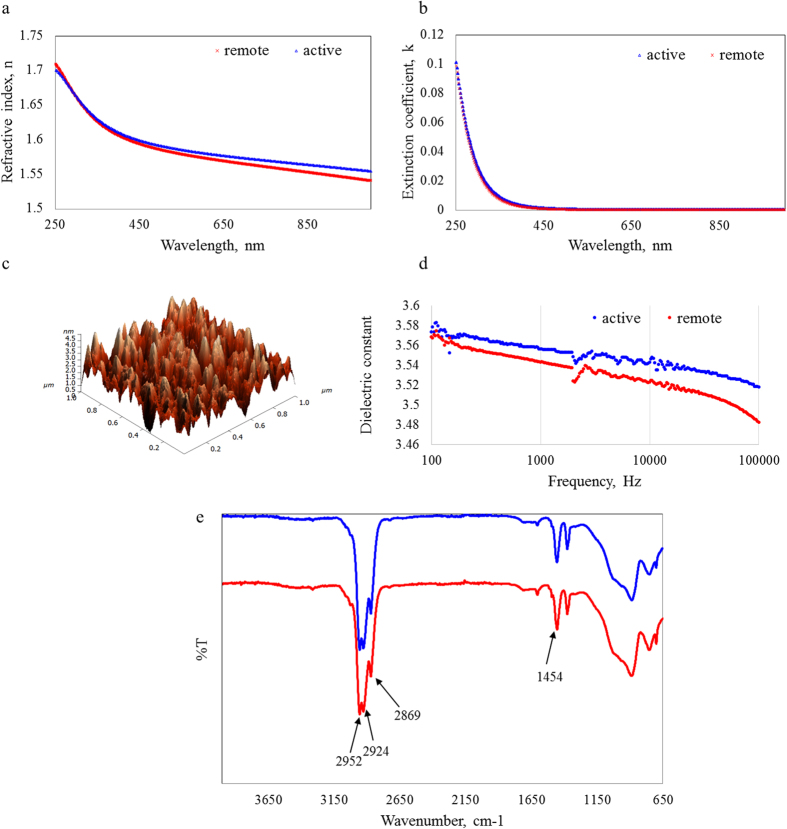
A comparison between material properties of cis−β−ocimene films fabricated in the active plasma region and outside of plasma glow at applied power of 50 W: (**a**) refractive index; (**b**) coefficient of extinction; (**c**) surface profile of film deposited outside of the plasma glow; (**d**) dielectric constant; (**e**) FTIR spectra.

The effect of substrate position on refractive index and extinction coefficient was not significant (Fig. 4). Samples were optically transparent above 450 nm. The refractive index was slightly lower in the 650−1000 nm wavelength region in the case of remote deposition samples. On the other hand, the optical band gap was slightly higher for samples fabricated outside of the plasma glow, at 2.89 eV compared to 2.83 eV for samples fabricated in the active plasma region at 50 W applied power. The dielectric constant was similar for the two types of samples, however, dielectric constant values began to drop off more rapidly at higher frequencies. This was attributed to slightly different polymer chemistry of films deposited in the active and remote plasma regions.

Samples deposited downstream from the active plasma region were characterised by a slightly higher contact angle, e.g. ~80.0° ± 2.2° and 76.5° ± 3.3° for the samples synthesised at 50 W applied power in the remote and active plasma regions, respectively. Examination of FTIR data showed no significant difference in terms of the nature or intensity of the peaks, suggesting that the position of the substrate had little effect on the chemical composition of the film. Substrate position can affect such film properties as film density, surface chemistry and surface roughness^58^. A significant change in film density would affect the topography of the sample, thereby varying its adhesion and contact angle properties. Deposition outside of the plasma glow region resulted in samples with similar maximum peak height, at 4.5 nm, significantly lower average and root mean square roughness, at 2.2 nm and 0.7 nm, respectively, compared to 4.4 nm, 2.7 nm, and 4 nm reported for samples deposited under the same applied power of 50 W in the region of plasma glow. The decrease in roughness was attributed to the absence of surface damage induced by direct ion bombardment. The surfaces of samples deposited downstream from the glow region were characterised by skewness of 0.1, suggestive of a surface with good symmetry of the deviations of a surface profile about the mean line. Coefficient of kurtosis value was significantly lower in the case of the samples deposited remotely, at 0.2 and 1.7 for remote and active depositions at 50 W applied power, respectively. Both values are below 3, indicating a distribution with fewer, less extreme outliers compared to Gaussian distribution.

### Thermal stability

**Figure 5:**
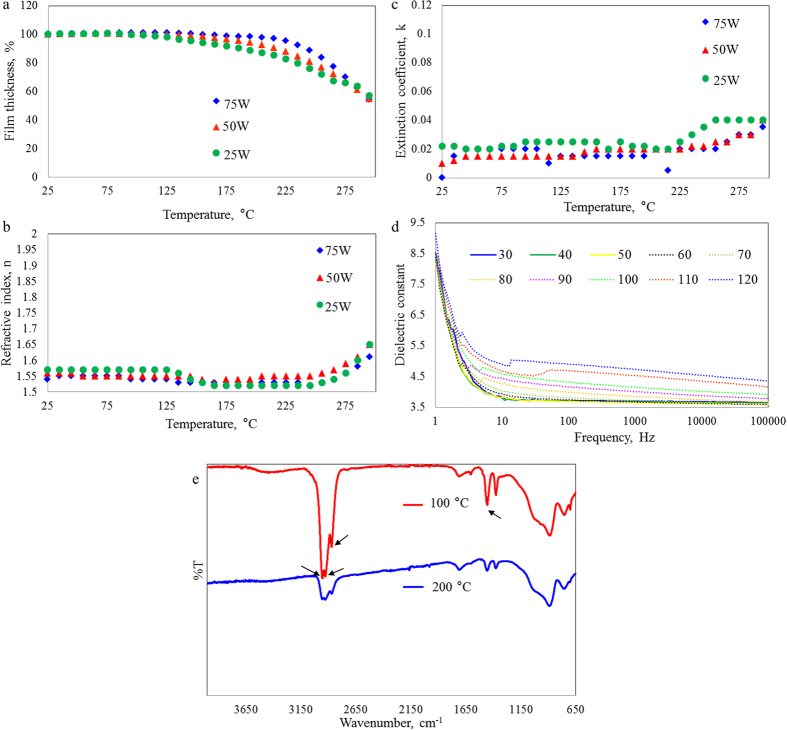
Thermal degradation behavior of cis−β−ocimene as a function of deposition power: (**a**) film thickness; (**b**) refractive index; and (**c**) extinction coefficient. (**d**) The effect of increasing temperature on dielectric constant of cis−β−ocimene thin film fabricated at 25 W. (**e**) The effect of temperature on FTIR spectra of cis−β−ocimene film deposited at 25 W.

Thermal stability of the material properties of cis−β−ocimene polymers was determined by measuring changes in the thickness, optical properties and dielectric constant of the films as functions of temperature. As shown in Fig. 5, film degradation commenced at 125, 165 and 205 °C for samples fabricated at 25, 50 and 75 W, respectively. Between 200 °C to 250 °C, the reduction in thickness becomes more prominent for all samples however the refractive index remains stable. After 250 °C, a phase change occurs and structural changes take place as characterised by the increasing refractive index and extinction coefficient^37^. Relatively higher onset temperature and lower degradation rate of samples fabricated at higher applied power may be attributed to higher degree of crosslinking characteristic of these films^16^. Given that the extinction coefficient remained ~0 and the refractive index remained stable within the 25−250 °C, cis−β−ocimene polymers can be used in devices requiring thermal processing, e.g. annealing or evaporation deposition, without detriment to their thickness or optical characteristics. Annealing to 405 °C resulted in almost complete decomposition of the films, with residual thickness ranging between 2.36 and 7.15%, comparable to residue levels reported for plasma-polymerised poly(methyl methacrylate) and other plasma polymers^59^,^60^.

**Figure 6:**
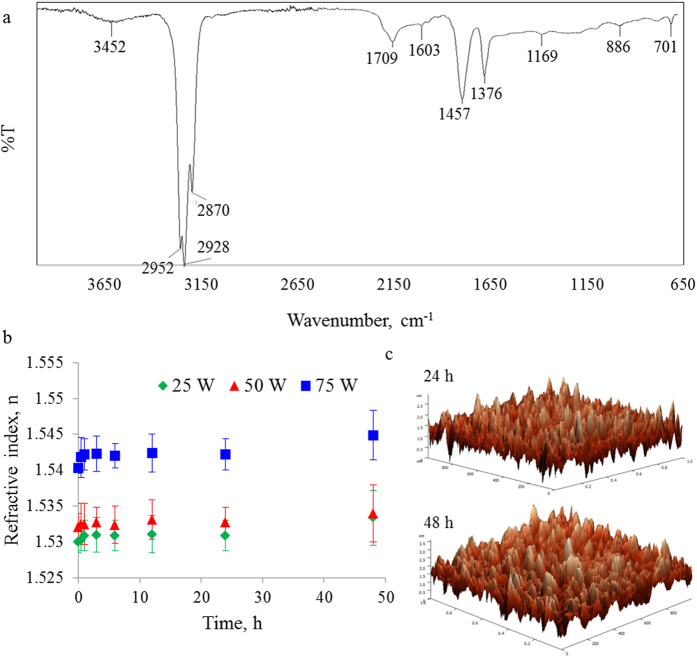
Material properties of cis−β−ocimene films after incubation in simulated body fluid: (**a**) FTIR spectrum of film fabricated at 50 W after 48 h of immersion; (**b**) refractive index of films fabricated at different applied power as a function of immersion time; (**c**) AFM images of cis−β−ocimene film surface (polymer deposited at 50 W) after 24 and 48 h of immersion.

In the low frequency range of 1 Hz−100 kHz, an LCR meter was used to measure the capacitance of the samples as they were heated to 120 °C and calculate the dielectric constant as a function of temperature. The operating range for OLED devices in consumer electronics is not expected to exceed this temperature. Figure 6 shows that above 50 °C, there is a clear increase in the dielectric constant across the entire frequency range. This increase was attributed to the higher temperatures disturbing the alignment of molecules in the films, producing molecules which are free to reorient in an external field^61^. Consequently, as the number of molecules free to move increase, so does the dielectric constant.

Optical and electrical properties of samples fabricated at the lowest power level experienced most changes with increasing temperature, the effect attributed to lower level of cross-linking in their molecular structure compared to films produced at higher applied power. In order to correlate changes in optical properties to molecular structure, ATR measurements were taken from samples heated to 100 °C, 200 °C and 300 °C (Fig. 5). Data acquired from the sample heated to 200 °C demonstrate a reduction in spectrum quality, attributed to both the decrease in film thickness and changes in the chemical structure of the polymer. It is worth to note that all experiments were conducted under ambient atmosphere, which, in conjunction with heating, may have contributed to an observed increase in the peak associated with carboxylic acid and other oxygen-containing moieties^62^. At 300 °C, substantial loss of thickness results in poorly-resolved peaks.

Due to the proportional increase of oxygen-containing groups in the material, its ability to polarise will become greater and as such the dielectric constant is expected to increase^63^. This is in agreement with experimental data, where cis−β−ocimene samples produced at 25 W are characterised by a higher concentration of hydroxyl as well as the highest dielectric constant amongst the samples. In terms of device application, an increased dielectric constant of cis−β−ocimene film at higher temperature means that when this film used as an interlayer in OLED devices, its efficiency will not be diminished when heated to operating temperature and may improve its performance.

Supplementary Figure S1 shows the effect of substrate position on the thermal stability of cis−β−ocimene films fabricated within and outside of the plasma glow region. Modelling of film thickness shows that polymers fabricated in the afterglow region of the plasma have higher onset degradation temperature. This is possibly due to the fact that remote conditions allow minimising excessive ion bombardment of the sample and plasma-associated heating of the substrate, thus limiting the number of free radicals that remain within and on the surface of the polymers. Deposition under different energy density conditions will affect the molecular structure of the film^58^. Polymers fabricated downstream did not experience a large increase in permittivity, and may therefore be more suitable for applications where property stability is desired.

### Biodegradation

Biointegration is rapidly emerging area of research that benefits from the unique advantages offered by organic materials and promises to revolutionise how biomedical services and medical care are delivered to patients. Biocompatible OLEDs have the potential to significantly advance biophotonics and optogenetics by providing customise light delivery methods for *in vitro* and *in vivo* modulation of cell and animal activity through highly-resolved spatiotemporal optical stimulation^6^. To achieve this objective, devices need to maintain their stability and efficacy when operated under aqueous conditions. Stability of cis−β−ocimene in aqueous environment was evaluated by immersing polymer thin films into simulated body fluid comprising of Na^+^ 142.0 mM, K^+^ 5.0 mM, Mg^2+^ 1.5 mM, Ca^2+^ 2.5 mM, HCO^3−^ 4.2 mM, Cl^−^ 147.8 mM, HPO_4_^2−^ 1.0 mM and SO_4_^2−^ 0.5 mM, and incubating them body temperature (~37 °C). After incubation, the samples were gently rinsed with double-distilled water and allowed to air dry. Their optical, surface and chemical properties were then evaluated using ellipsometry, AFM and FTIR, respectively. These results are summarised in Fig. 6.

FTIR spectra of the films did not change significantly as a result of incubation, with peaks retaining their position and intensity. The peak associated with −OH group increased slightly, likely due to the adsorption of water molecules on the surface of the polymer films. Independent of deposition power, refractive index and coefficient of extinction also remained relatively stable, with a slight, not statistically significant increase in the refractive index observed after 48 h of immersion. Visual examination of the samples confirmed their optical transparency. In terms of surface morphology, the samples remained smooth, with the average roughness of 2.5−3.5 nm, and RMS roughness of less than 1 nm.

### Biocompatibility

Biointegration of organic electronic devices with living tissue is an exciting and highly-topical area of research. To assess the potential use of cis−β−ocimene polymer coating as encapsulating coating for implantable electronic devices, non-adherent THP–1 cells and adherent mouse macrophage cells were incubated in the presence of cis−β−ocimene polymer coatings *in vitro*, and their viability, proliferation, and size, attachment and morphology (in the case of macrophages) were evaluated. Previous investigations have demonstrated excellent cytocompatibility of thin films fabricated using plasma polymerisation from other essential oil-based precursors, e.g. terpinene-4-ol^23^,^64^,^65^,^66^.

The results of cell studies are summarised in Table 2. After 24 h of incubation, the viability of THP–1 cells was similar for wells containing inert unmodified glass and the polymer samples, independent of the deposition power used during polymer synthesis. Cell numbers were not statistically different when incubated in wells containing unmodified glass and polymer films after 24 h and 48 h of incubation.

**Figure 7:**
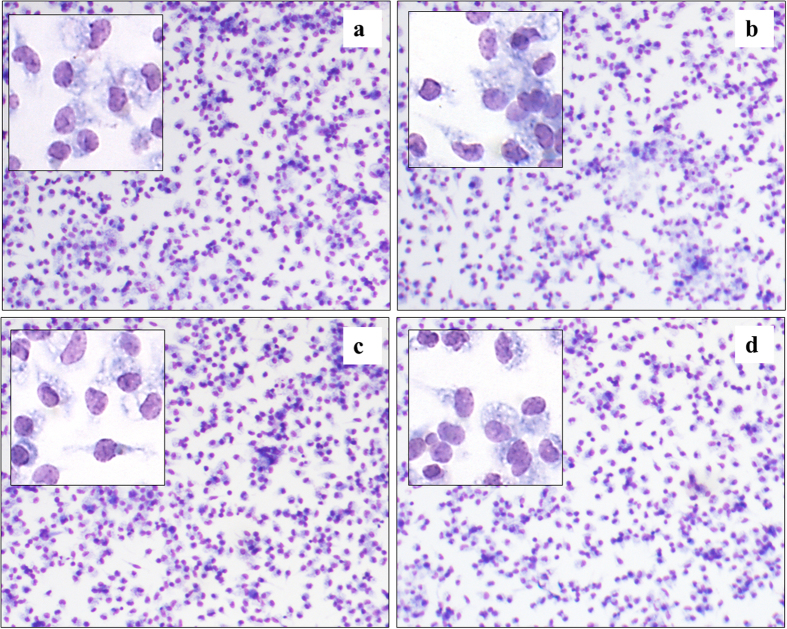
Optical micrographs of mouse macrophage cells that were cultured on the surface of (**a**) inert glass, and glass coated with the polymer fabricated at (**b**) 25 W, (**c**) 50 W, and (**d**) 75 W for 48 h. Image magnification × 10, inset magnification × 40.

Similarly, the viability and attachment behaviour of mouse macrophage cells was similar across all sample types after 24 h of incubation (Fig. 7). Optical visualisation of attached cells showed healthy morphology characterised by a flattened well–spread shape. There was no statistically significant difference between the number of cells observed on glass control and polymer-coated samples, or within polymers fabricated under different RF power conditions. For cells incubated for 48 h, the cell monolayers were washed after 24 h to remove cells that failed to attach, and fresh culture media was added. The cells were left for additional 24 h under the same incubation conditions and visualised. After 48 h, the number of attached viable cells was similar across sample types, and not statistically different from cell number recorded after 24 h of incubation. To investigate the effect of exotoxin stimulation on the attachment behaviour of mouse macrophages, some of the wells containing glass slides and polymer-coated samples were supplemented with *Escherichia coli* lipopolysaccharide (LPS). In mouse macrophages, LPS behaves as a prototypical endotoxin, binding to the CD14/TLR4/MD2 receptor complex, and promoting the secretion of pro-inflammatory cytokines, reactive oxygen and nitrogen species, e.g. superoxide and NO, and eicosanoids. LPS was added at the concentration of 50 ng/ml. Cell count on samples exposed to LPS was not significantly different between the sample types, however they were significantly lower than in the case of cells that received no stimulation.

## Conclusion

Cis−β−ocimene thin films are optically transparent, smooth (*R*_*q*_ < 1 nm) and defect free, with a refractive index of ~1.58 at 600 nm, an optical band gap of ~2.85 eV, and dielectric constant of 3.5−3.6 at 1 kHz. The surfaces of these films are sufficiently wettable for solution processed electronics, with water contact angle of 76−80°, and biocompatible with non-adherent THP–1 cells and adherent mouse macrophage cells, including LPS-stimulated macrophages. These properties remain stable upon heating up to 200 °C, making them compatible with annealing and thermal deposition techniques. Heating of the sample increases the dielectric constant, from 3.7 (25 °C) to 4.7 (120 °C) at 1 kHz for polymer fabricated at 25 W. These films are also stable under aqueous conditions, maintaining their material properties after 48 h of immersion into simulated body fluid, and hence can potentially be incorporated as dielectric or encapsulating layers into devices used *in vitro* and *in vivo*.

## Additional Information

**How to cite this article**: Bazaka, K. *et al*. Plant-derived cis-β-ocimene as a precursor for biocompatible, transparent, thermally-stable dielectric and encapsulating layers for organic electronics. *Sci. Rep.* **6**, 38571; doi: 10.1038/srep38571 (2016).

**Publisher's note:** Springer Nature remains neutral with regard to jurisdictional claims in published maps and institutional affiliations.

## Supplementary Material

Supplementary Information

## Figures and Tables

**Table 1:** Surface parameters of cis−β−ocimene polymers as a function of applied RF power.

	Applied power, W		
	25 W	50 W	75 W
*1 μm* × *1 μm*			
Maximum peak height (nm)	7.7	4.4	5.6
Average roughness (nm)	3.3	2.7	3.1
Root Mean Square (nm)	0.9	0.4	0.5
Surface skewness	0.5	0.1	0.6
Coefficient of kurtosis	0.8	1.7	4.1
*10 μm* × *10 μm*			
Maximum peak height (nm)	20.8	15.7	9.6
Average roughness (nm)	3.8	1.9	3.0
Root Mean Square (nm)	1.9	1.2	1.8
Surface skewness	3.8	3.9	3.6
Coefficient of kurtosis	1.0	1.2	0.8
*Contact angle, °*			
Water	76.0 ± 0.8	76.5 ± 2.2	78.9 ± 1.8
Ethylene glycol	39.1 ± 1.7	42.5 ± 1.2	46.1 ± 0.9
Diiodomethane	39.5 ± 0.7	39.6 ± 0.9	39.7 ± 1.1
*Surface energy, mN/m*			
γ_S_	30.6 ± 2.1	32.2 ± 1.0	34.6 ± 1.1

**Table 2:** Cell−surface interactions: THP–1 cell viability and proliferation and mouse macrophage attachment after incubation for 24 and 48 hours at 37 °C and 5% CO_2_.

	Control	Polymer		
		25 W	50 W	75 W
*THP−1*				
cell number, ×10 ^6^ cells/m*				
24 h	1.12 ± 0.08	1.08 ± 0.11	0.99 ± 0.18	1.01 ± 0.13
48 h	1.92 ± 0.21	1.94 ± 0.26	1.89 ± 0.18	1.91 ± 0.31
viability, %				
24 h	97.1 ± 1.1	96.8 ± 0.9	97.2 ± 1.7	96.0 ± 1.8
48 h	94.2 ± 0.9	95.1 ± 1.1	93.9 ± 1.6	94.1 ± 0.7
*Mouse macrophages*				
cell number per visualised area				
24 h	232.1 ± 22.7	229.2 ± 28.1	241.5 ± 31.2	219.9 ± 21.1
48 h	221.7 ± 37.1	241.1 ± 29.5	231.6 ± 29.3	229.5 ± 31.7
LPS	172.1 ± 33.5	182.6 ± 17.9	192.4 ± 23.8	187.2 ± 25.8

^*^Initial cell count 0.5 × 10 ^6 ^cells/ml, viability 95%.
