# Supplementary material for: Plant-derived cis-β-ocimene as a precursor for biocompatible, transparent, thermally-stable dielectric and encapsulating layers for organic electronics
Source: Sci Rep. 2016 Dec 9;6:38571. doi: 10.1038/srep38571 (PMC5146940; doi:10.1038/srep38571)
Supplement: Supplementary Information [file srep38571-s1.pdf]

Plant-derived cis-β-ocimene as a precursor for biocompatible, transparent, thermally-stable dielectric and encapsulating layers for organic electronics

Kateryna Bazaka[1,2,3,4,5]* , Ryan Destefani[2], Mohan V. Jacob[2]*

Supplementary Information

Table S1. Identification of functional groups present in FTIR spectra of cis−β−ocimene thin films.

Wavenumber, cm^{-1}	Assignment
3200−3600	Hydroxyl O−H
2952	Methyl asymmetric C−H stretch
2924	Methylene asymmetric C−H stretch
2869	Methyl symmetric C−H stretch
1706	Carbonyl/carboxylic acid
1603	Alkene conjugated C=C vibration
1492	Methylene symmetric C−H stretch
1454	Methyl asymmetric C−H stretch
1376	Methyl symmetric C−H stretch
1300−700	Skeletal C−C vibration

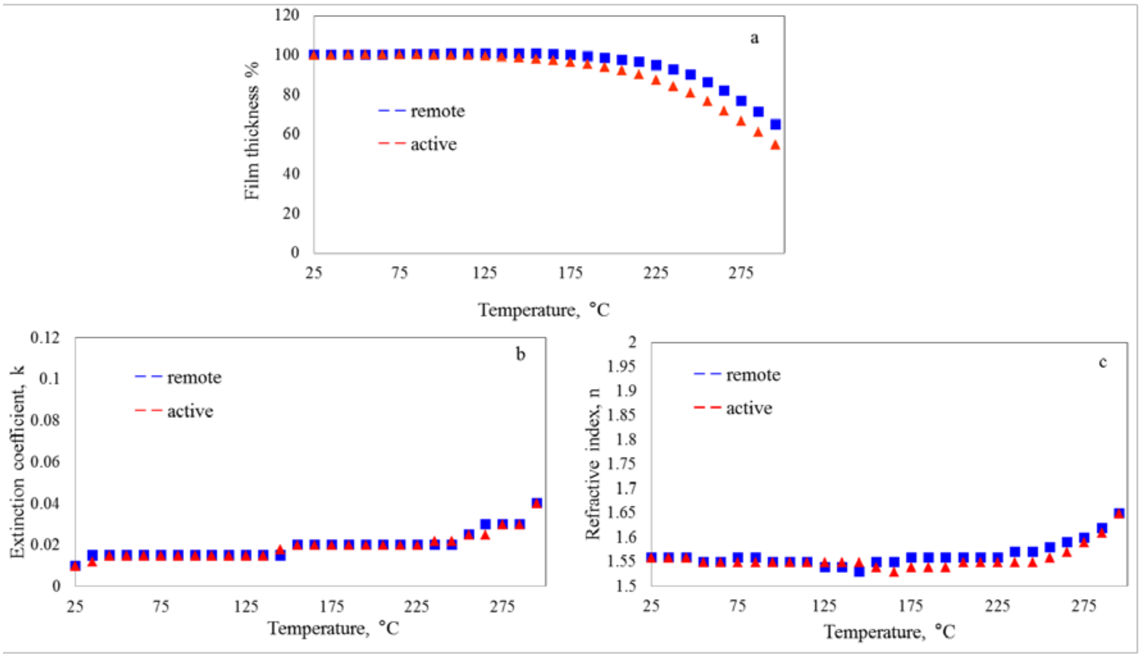

Figure S1. The effect of substrate location on thermal stability of cis−β−ocimene polymer thin films: **a.** film thickness; **b.** extinction coefficient; and **c.** refractive index.
